# Hypoxia-induced sulfide:quinone oxidoreductase suppresses ferroptosis and promotes gemcitabine resistance through mitochondrial coenzyme Q reduction in pancreatic ductal adenocarcinoma

**DOI:** 10.3389/fcell.2026.1971375

**Published:** 2026-10-19

**Authors:** Guixiang Huang, Xiang An, Tongling Yuan, DongXu Liao

**Affiliations:** 1 Department of Emergency Surgery, Sichuan Provincial People’s Hospital, School of Medicine, University of Electronic Science and Technology of China, Chengdu, Sichuan, China; 2 Department of Hepatobiliary and Pancreatic Surgery, Sichuan Provincial People’s Hospital, School of Medicine, University of Electronic Science and Technology of China, Chengdu, Sichuan, China; 3 General Practice Center, Sichuan Provincial People’s Hospital, School of Medicine, University of Electronic Science and Technology of China, Chengdu, Sichuan, China

**Keywords:** coenzyme Q, ferroptosis, gemcitabine resistance, hypoxia, lipid peroxidation, pancreatic ductal adenocarcinoma, patient-derived organoids, sulfide:quinone oxidoreductase

## Abstract

**Background:**

Pancreatic ductal adenocarcinoma (PDAC) is highly resistant to gemcitabine and arises in a profoundly hypoxic tumor microenvironment. However, the molecular link between hypoxic adaptation and ferroptosis evasion remains incompletely understood. We investigated sulfide:quinone oxidoreductase (SQOR), a mitochondrial enzyme that couples hydrogen sulfide oxidation to coenzyme Q (CoQ) reduction, as a hypoxia-responsive regulator of ferroptosis sensitivity.

**Methods:**

A tissue microarray from 102 patients with PDAC was analyzed for hypoxia markers, SQOR, 4-hydroxynonenal (4-HNE), glutathione peroxidase 4 (GPX4), and ferroptosis suppressor protein 1 (FSP1), together with treatment response and survival. Mechanistic studies combined hypoxia time-course experiments with SQOR knockdown, wild-type overexpression, and a catalytically inactive mutant; electron-donor testing; CoQ redox and lipidomic profiling; GPX4/FSP1/dihydroorotate dehydrogenase (DHODH) epistasis; and death-mode rescue assays. Therapeutic relevance was evaluated in gemcitabine-resistant cell lines, eight patient-derived organoid lines, and xenograft models.

**Results:**

SQOR protein H-scores showed moderate positive associations with CAIX and HIF-1α (Spearman ρ = 0.473 and 0.438; BH-adjusted q = 3.92544 × 10^−6^ and 1.57881 × 10^−5^, respectively) and an inverse association with the lipid-peroxidation marker 4-hydroxynonenal (ρ = −0.465; q = 4.13981 × 10^−6^) and higher SQOR abundance was associated with shorter overall survival after multivariable adjustment for GPX4 and FSP1 (adjusted hazard ratio, 1.87). Hypoxia increased SQOR expression and the CoQH_2_/total CoQ ratio while suppressing lipid peroxidation; these effects required SQOR catalytic activity, and donor-perturbation experiments implicated H_2_S more strongly than selenium metabolism under the tested conditions. SQOR loss increased relative signals annotated as oxidized arachidonoyl- and adrenoyl-phosphatidylethanolamines and enhanced ferroptosis sensitivity. SQOR loss synergized with inhibition of GPX4, FSP1, and DHODH, supporting a nonredundant contribution to ferroptosis control; pharmacological rescue was more pronounced with ferroptosis-directed agents than with a caspase inhibitor. Combining the putative SQOR inhibitor with ferroptosis induction lowered gemcitabine IC_50_ values in resistant models, without restoring parental sensitivity.

**Conclusion:**

The tested PDAC models support SQOR as a hypoxia-responsive, catalysis-dependent contributor to mitochondrial ferroptosis defense and gemcitabine resistance. The tissue correlations are associative and do not establish that hypoxia suppresses lipid peroxidation through SQOR in patients. These findings support further preclinical evaluation of SQOR-directed strategies to improve gemcitabine sensitivity in PDAC.

## 1 Introduction

Despite sustained therapeutic development, pancreatic ductal adenocarcinoma (PDAC) remains one of the most lethal solid malignancies, with a five-year relative survival rate of approximately 13% (Siegel et al., 2025). Gemcitabine became a standard treatment after producing a modest improvement in clinical benefit and survival over fluorouracil (Burris et al., 1997). Although FOLFIRINOX and newer gemcitabine-based combinations have extended survival in selected patients with metastatic disease, durable disease control remains uncommon (Conroy et al., 2011; Nichetti et al., 2024). Intrinsic and acquired resistance to gemcitabine therefore continue to constrain treatment efficacy across disease stages (Amrutkar and Gladhaug, 2017).

This resistant phenotype is shaped in part by the distinctive PDAC microenvironment. Dense desmoplastic stroma and poor vascularization restrict tissue perfusion and drug delivery (Olive et al., 2009), while direct oxygen measurements have confirmed marked hypoxia within human pancreatic tumors (Koong et al., 2000). Hypoxia is not merely a consequence of tumor growth; it can reprogram cancer-cell metabolism and promote chemoresistance. Recent studies have linked the hypoxic and acidic PDAC microenvironment to specific resistance circuitry (Ding J. et al., 2025), while three-dimensional tumor growth can induce metabolic programs that protect cancer cells from oxidative cell death (Seo et al., 2025). The molecular pathways connecting hypoxic adaptation to drug resistance, however, remain incompletely defined.

Ferroptosis is an iron-dependent, non-apoptotic form of regulated cell death driven by phospholipid peroxidation (Dixon et al., 2012). It is particularly relevant to treatment resistance because therapy-resistant mesenchymal cell states can acquire a selective dependence on glutathione peroxidase 4 (GPX4)-mediated lipid-peroxide detoxification (Viswanathan et al., 2017). PDAC also displays a preclinical ferroptotic vulnerability: cysteine depletion induces ferroptosis and restrains tumor growth in genetically engineered mouse models (Badgley et al., 2020). Conversely, PDAC cells can evade ferroptosis by increasing GPX4 expression or activating antioxidant programs such as the HSP90–KEAP1–NRF2 axis (Liu et al., 2024; Luo et al., 2024). These findings suggest that ferroptosis resistance may contribute to the persistence of PDAC cells under therapeutic stress.

Several partially independent systems determine the ferroptosis threshold. The glutathione-dependent lipid hydroperoxidase GPX4 constitutes the canonical defense against phospholipid peroxidation (Yang et al., 2014). At non-mitochondrial membranes, ferroptosis suppressor protein 1 (FSP1) reduces CoQ to its radical-trapping antioxidant form, CoQH_2_, and acts in parallel with GPX4 (Bersuker et al., 2019; Doll et al., 2019). Within mitochondria, dihydroorotate dehydrogenase (DHODH) provides a separate CoQ-dependent defense against lipid peroxidation (Mao et al., 2021). Ferroptosis sensitivity also depends on membrane composition: ACSL4 enriches membranes in oxidation-prone polyunsaturated fatty-acid phospholipids, whereas ACSL3-dependent incorporation of monounsaturated fatty acids promotes a ferroptosis-resistant state (Doll et al., 2017; Magtanong et al., 2019). Thus, both antioxidant capacity and lipid-substrate availability can be metabolically reprogrammed to alter ferroptosis sensitivity. How hypoxia engages mitochondrial CoQ redox control in PDAC remains unclear.

Sulfide:quinone oxidoreductase (SQOR) is a flavoprotein of the inner mitochondrial membrane that catalyzes the first committed step of H_2_S oxidation and transfers the resulting electrons to CoQ (Jackson et al., 2012; Landry et al., 2017). This biochemical position makes SQOR a plausible link between hypoxic metabolic adaptation, CoQH_2_ availability, and suppression of lipid peroxidation. Recent work showed that selenium metabolism can also support SQOR-dependent ubiquinone reduction and ferroptosis suppression (Lee et al., 2024), and SQOR has consequently been proposed as a broader regulator of redox homeostasis (Peng et al., 2025). Whether SQOR controls ferroptosis in hypoxic PDAC, which physiological electron donor predominates in this setting, and how SQOR relates functionally to GPX4, FSP1, and DHODH remain unresolved.

Recent studies in PDAC further support a therapeutic link among metabolic dependencies, ferroptosis, and chemotherapy response. Disrupting glutamine metabolism can expose a GPX4-dependent ferroptotic vulnerability (Shen et al., 2025), while pharmacological induction of ferroptosis can enhance gemcitabine activity (Li J. et al., 2025). We therefore hypothesized that hypoxia-induced SQOR increases the CoQH_2_/CoQ_total_ ratio, limits phospholipid peroxidation, and raises the ferroptosis threshold, thereby contributing to gemcitabine resistance. We further hypothesized that the SQOR–CoQ axis forms a partially independent, rather than fully redundant, branch of the ferroptosis-defense network alongside GPX4, FSP1, and DHODH. To test these hypotheses, we integrated a 102-patient tissue microarray with catalytic-mutant analysis, electron-donor validation, lipidomics, pathway epistasis, death-mode rescue, and therapeutic studies in gemcitabine-resistant cells, patient-derived organoids, and xenografts.

## 2 Materials and methods

### 2.1 Study design

This four-stage translational study investigated the relationships among tumor hypoxia, SQOR-mediated mitochondrial CoQ/CoQH_2_ redox cycling, ferroptosis resistance, and gemcitabine resistance in pancreatic ductal adenocarcinoma (PDAC). Stage 1 comprised a prespecified patient-level analysis of 102 PDAC cases. Duplicate tissue microarray (TMA) cores were averaged for each patient before analysis; thus, the patient, rather than the core, field, or cell, was the statistical unit for biomarker correlations, treatment-response analyses, and survival analyses. Stage 2 used PANC-1 and MIA PaCa-2 models for hypoxia time-course and SQOR genetic-manipulation experiments. Stage 3 comprised pharmacological and genetic pathway-epistasis experiments. Stage 4 used resistant cell lines, eight independent patient-derived organoid (PDO) lines, and two separate xenograft cohorts for tumor-growth/terminal-weight and survival endpoints.

In Stage 2, two-dimensional monolayer and three-dimensional spheroid hypoxia models were established in two PDAC cell lines with KRAS-mutant backgrounds and distinct phenotypic characteristics. SQOR knockdown, lentiviral overexpression, and expression of a catalytically dead point mutant were used to distinguish the enzymatic contribution of SQOR to CoQ reduction from effects attributable to protein abundance and to determine whether SQOR activity regulates mitochondrial and whole-cell lipid peroxidation. The physiological electron donor supporting SQOR catalysis (H_2_S or H_2_Se) was tested experimentally rather than assumed.

In Stage 3, SQOR manipulation was combined with pharmacological inhibition of GPX4, FSP1, and DHODH in pathway-epistasis experiments, together with ferroptosis- and apoptosis-specific death-modality rescue assays. These experiments tested whether the SQOR–CoQ axis makes a nonredundant contribution to the mitochondrial ferroptosis-defense network or is functionally redundant with established pathways. They assessed whether the viability loss was consistent with a substantial ferroptotic contribution; rescue assays were not used to assign mutually exclusive death fractions.

Stage 4 translated these mechanistic findings into therapeutic-resistance models. Gemcitabine sensitivity was evaluated in parental and resistant PANC-1 and MIA PaCa-2 cells, eight independent PDO lines (four SQOR-high and four SQOR-low), and subcutaneous xenografts generated from gemcitabine-resistant PDAC cells. Tumor-growth/terminal-weight experiments (n = 6 mice per group) and survival experiments (n = 7 mice per group) were performed in separate randomized cohorts. Technical wells, microscopic fields, and duplicate tissue cores were averaged within their respective biological units and were not counted as independent n values.

### 2.2 Patient cohort and tissue microarray construction

This retrospective cohort included 102 patients with histologically confirmed PDAC who underwent surgical resection or biopsy followed by gemcitabine-based chemotherapy and whose samples were available in institutional archives. Eligibility required formalin-fixed, paraffin-embedded (FFPE) tissue with adequate tumor content (>70% tumor cellularity), complete clinicopathological documentation, and at least 4 months of follow-up. Patients were excluded if they had received neoadjuvant therapy before tissue acquisition or had incomplete treatment-response documentation. Two board-certified pathologists independently reviewed hematoxylin–eosin-stained sections to identify representative viable tumor regions while excluding necrotic and hemorrhagic areas; disagreements were resolved by consensus. Duplicate 1.5-mm cores from each donor block were transferred to recipient TMA blocks using a semi-automated tissue arrayer, and 4-µm sections were subsequently cut onto charged slides for staining. Baseline cohort characteristics are summarized in Table 1.

**TABLE 1:** Clinicopathological characteristics of the study cohort (N = 102).

Characteristic	Value
Age, years (mean ± SD)	61.4 ± 9.2
Sex — Male/Female	58 (56.9)/44 (43.1)
Tumor location — Head/Body-tail	66 (64.7)/36 (35.3)
Tumor size, cm (mean ± SD)	3.6 ± 1.4
Differentiation — Well/moderately differentiated/Poorly differentiated	47 (46.1)/55 (53.9)
TNM stage — I–II/III–IV	61 (59.8)/41 (40.2)
Lymph node metastasis — Negative/Positive	49 (48.0)/53 (52.0)
Perineural invasion — Absent/Present	54 (52.9)/48 (47.1)
CA19-9 — ≤ 37 U/mL/> 37 U/mL	22 (21.6)/80 (78.4)
Chemotherapy regimen — Gemcitabine monotherapy/Gemcitabine-based combination	41 (40.2)/61 (59.8)
Mixed response — Responder/Non-responder	44 (43.1)/58 (56.9)
Median follow-up, months (range)	26.5 (4–48)

Treatment response was classified in all 102 patients using the study-specific hierarchical algorithm. Patients with measurable disease and evaluable imaging were classified as responders for complete or partial response, and non-responders for stable or progressive disease, according to RECIST 1.1 (Eisenhauer et al., 2009). For postoperative patients without measurable disease, the criterion was a ≥50% decrease in CA19-9 from baseline within the first two chemotherapy cycles. When available assessments were discordant, RECIST classification took precedence. Of the 102 patients, 63 had measurable disease and evaluable imaging and were classified using RECIST 1.1: 2 complete responses, 23 partial responses, 21 cases of stable disease, and 17 cases of progressive disease. The remaining 39 postoperative patients without measurable disease were classified using the study-specific CA19-9 criterion; 19 showed a ≥50% decline within the first two chemotherapy cycles, and 20 did not meet this threshold. The resulting exploratory mixed endpoint comprised 44 responders and 58 non-responders. It combines radiological tumor change with a biochemical change in a different clinical setting and is therefore treated as an exploratory response endpoint, not a RECIST objective response rate. The ≥50% CA19-9 threshold is a study-specific definition; the MPACT analysis supports the complementary prognostic information in CA19-9 decline but does not validate that threshold in postoperative disease (Chiorean et al., 2016).

### 2.3 Immunohistochemical staining panel

Serial 4-µm TMA sections were deparaffinized, rehydrated, and subjected to heat-induced epitope retrieval in citrate buffer (pH 6.0) or EDTA buffer (pH 9.0), as recommended by the antibody manufacturer, in a pressure cooker at 121 °C for 15 min. Endogenous peroxidase activity was quenched with 3% hydrogen peroxide, after which nonspecific protein binding was blocked with 5% normal goat serum for 30 min at room temperature. Sections were incubated overnight at 4 °C with primary antibodies against carbonic anhydrase IX (CAIX), hypoxia-inducible factor 1α (HIF-1α), SQOR, 4-hydroxynonenal (4-HNE), GPX4, and FSP1 at manufacturer-validated working dilutions. Species-appropriate HRP-conjugated secondary antibodies were then applied for 30 min at room temperature, followed by DAB development for a standardized duration. Slides were counterstained with Mayer’s hematoxylin, dehydrated, and mounted. Negative controls lacking the primary antibody and positive-control tissues with known marker expression were included in every staining batch.

Before cohort-wide staining, SQOR antibody specificity was independently validated by parallel immunohistochemical and Western blot analyses of FFPE-embedded SQOR-knockdown, SQOR-overexpressing, and wild-type cell pellets. Concordance between staining intensity and protein abundance confirmed antibody specificity.

Figure 1A depicts the clinical discovery stage, in which duplicate TMA cores from 102 PDAC cases were collapsed into one patient-level H-score per marker before correlation, chemotherapy-response, and survival analyses. This approach prevented tissue cores and microscopic fields from being treated as independent observations. Figure 1B summarizes the mechanistic stage: PANC-1 and MIA PaCa-2 cells underwent a 0–48-h hypoxia time course, followed by shSQOR, wild-type SQOR overexpression, or catalytically dead SQOR manipulation. CoQH_2_/total CoQ, whole-cell and mitochondrial lipid ROS, and oxidized phospholipids were assessed at the prespecified 24-h functional time point. Figure 1C links mechanism to function through shSQOR combinations with GPX4, FSP1, and DHODH inhibitors, orthogonal death-mode rescue assays, and validation in gemcitabine-resistant cells, eight independent PDO lines, and separate xenograft efficacy and survival cohorts. The workflow therefore proceeds from clinical association through catalytic validation and pathway positioning to therapeutic translation; the schematic itself does not represent outcome data.

**FIGURE 1:**
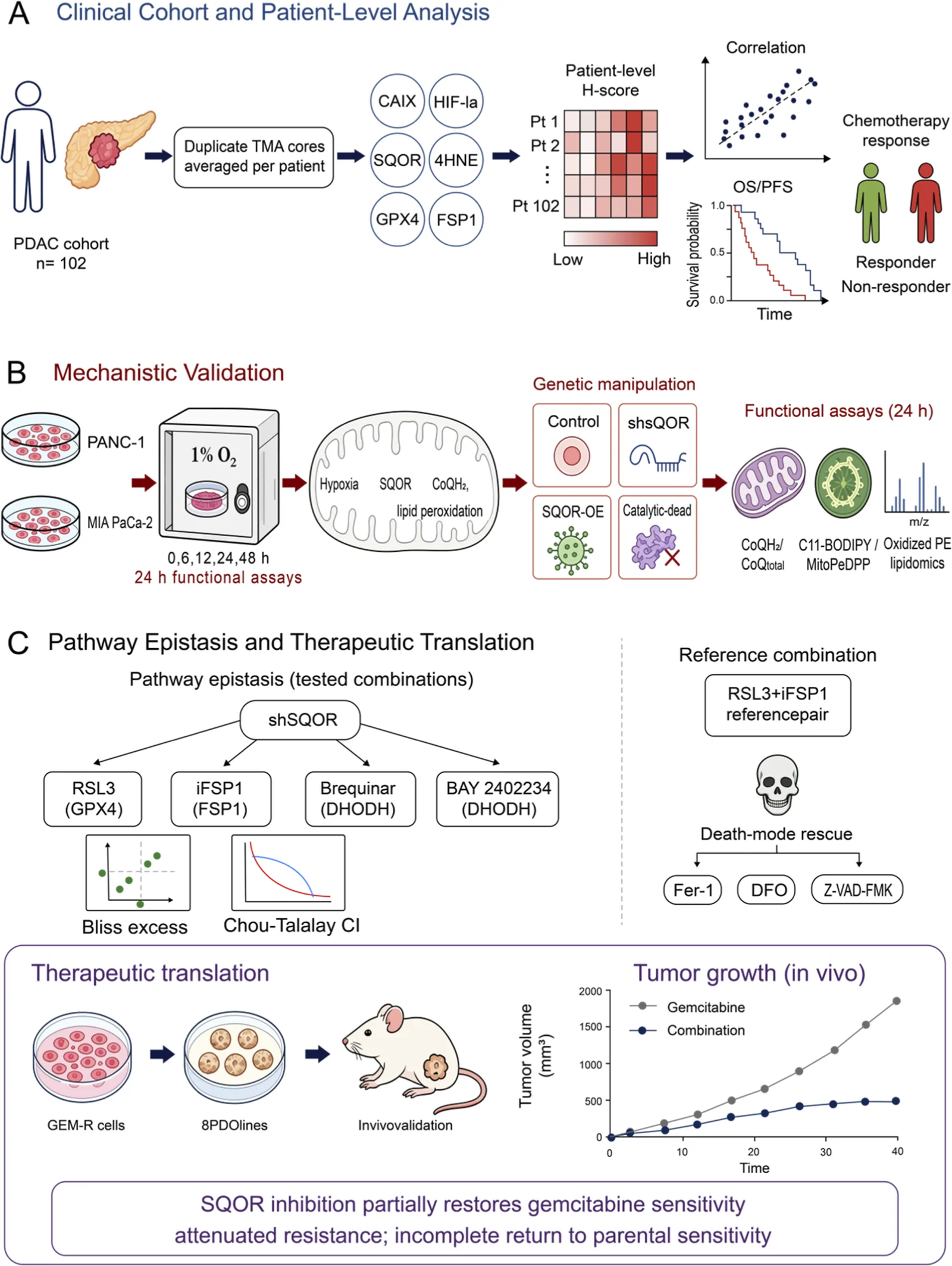
Overall study workflow integrating clinical, mechanistic, and functional modules. **(A)** Clinical cohort construction and patient-level biomarker, response, and survival analyses. **(B)** Hypoxia induction, SQOR genetic manipulation, and CoQ/lipid-peroxidation readouts. **(C)** shSQOR-centered pathway epistasis, death-mode rescue, and therapeutic validation in resistant cells, PDOs, and xenografts.

### 2.4 Digital quantification with QuPath

Stained slides were scanned at 20× magnification using a whole-slide digital scanner and analyzed in QuPath v0.4.4 (Bankhead et al., 2017). A pathologist blinded to clinical outcomes manually annotated tumor regions while excluding stromal, necrotic, and normal ductal areas. Positive-cell detection classified staining intensity as negative (0), weak (1+), moderate (2+), or strong (3+) and generated an H-score (Σ[intensity × percentage of positive cells]; range, 0–300). H-scores from duplicate TMA cores were averaged to obtain one value per marker per patient; all clinical correlation analyses therefore used n = 102 patient-level observations. Two independent blinded scorers evaluated a randomly selected 15% subsample, and an intraclass correlation coefficient >0.85 was required before automated scores were accepted. X-tile analysis using overall survival as the optimization endpoint identified an SQOR H-score cutoff of 156. Patients with an H-score >156 were classified as SQOR-high (n = 47), whereas those with an H-score ≤156 were classified as SQOR-low (n = 55). This grouping was applied without modification in the response and survival analyses (Camp et al., 2004). Because the outcome-optimized threshold was selected and evaluated in the same cohort, the high/low comparisons are exploratory and may overestimate prognostic separation. The threshold is not an independently validated clinical cutoff. Reported survival P values and confidence intervals are conditional on the selected grouping and do not incorporate cutoff-selection uncertainty (Altman et al., 2012).

### 2.5 Clinical correlation and survival analysis

Spearman rank correlation (ρ) was used for all pairwise patient-level associations among the CAIX, HIF-1α, SQOR, 4-HNE, GPX4, and FSP1 H-scores (n = 102), with Benjamini–Hochberg false-discovery-rate correction across the 15 pairwise tests. These analyses concern immunohistochemical protein-marker H-scores, not correlations between gene transcripts. Correlation coefficients describe the direction and magnitude of association; statistical significance does not establish a strong association or a causal relationship. The association between SQOR group and chemotherapy response was assessed using a Pearson chi-square test of association for the observed 2 × 2 table (SQOR-low: 30 responders and 25 non-responders; SQOR-high: 14 responders and 33 non-responders). Overall survival (OS) was measured from the first gemcitabine dose to death from any cause. Progression-free survival (PFS) was measured from the first gemcitabine dose to radiological recurrence or progression, or death. Kaplan–Meier curves were compared using two-sided log-rank tests. Cox proportional-hazards Model 1 adjusted for TNM stage, differentiation, lymph-node status, CA19-9 category, and chemotherapy regimen; Model 2 additionally included GPX4 and FSP1 expression. Proportional-hazards assumptions were assessed using Schoenfeld residuals. Model 1 conditions on indicators of disease extent, histological differentiation, serum CA19-9, and regimen; Model 2 examines the SQOR association after additional conditioning on GPX4 and FSP1. With the binary coding in Table 2, the models contain six and eight regression coefficients, respectively, including SQOR. This comparison assesses the association after adjustment for the included variables; it does not demonstrate adequate control of all clinical confounding, absence of overfitting, or a treatment-by-SQOR interaction.

**TABLE 2:** Prespecified Cox model estimates for overall survival (complete-case N = 102).

Model/variable	HR	95% CI	P value
Model 1 — SQOR expression (high vs. low)	2.14	1.32–3.47	0.002
Model 2 — SQOR expression (high vs. low; additionally adjusted for GPX4/FSP1)	1.87	1.11–3.15	0.019
Model 1 — TNM stage (III–IV vs. I–II)	2.63	1.58–4.38	1.99 × 10^−4^
Model 1 — Differentiation (poor vs. well-moderate)	1.58	0.97–2.57	0.066
Model 1 — Lymph-node metastasis (positive vs. negative)	1.79	1.08–2.97	0.024
Model 1 — CA19-9 (>37 vs. ≤37 U/mL)	1.42	0.79–2.55	0.239
Model 1 — Chemotherapy regimen (monotherapy vs. combination)	1.31	0.81–2.12	0.269
Model 2 — GPX4 expression (high vs. low)	0.71	0.44–1.15	0.164
Model 2 — FSP1 expression (high vs. low)	0.68	0.41–1.13	0.135

Model 1 adjusted for TNM stage, differentiation, lymph-node status, CA19-9 category, and chemotherapy regimen; Model 2 additionally included GPX4 and FSP1. The two SQOR estimates come from separate models, not a single simultaneous model. Each model included 102 patients and 75 deaths. Model 1 contained six fitted parameters including SQOR (12.5 events per parameter), and Model 2 contained eight (9.4 events per parameter). Global Schoenfeld tests did not provide evidence against proportional hazards (P = 0.64 and P = 0.61, respectively). These exploratory estimates do not constitute a validated prognostic model.

### 2.6 Cell culture and hypoxia modeling

The human PDAC cell lines PANC-1 and MIA PaCa-2, both harboring KRAS codon 12 mutations but exhibiting distinct phenotypic characteristics, were obtained from authenticated repositories and confirmed to be mycoplasma-negative. Cells were cultured in DMEM supplemented with 10% fetal bovine serum and 1% penicillin–streptomycin at 37 °C in a humidified incubator containing 5% CO_2_. Hypoxia was induced in a tri-gas workstation set to 1% O_2_ and 5% CO_2_, with N_2_ as the balance gas; parallel cultures maintained at 21% O_2_ served as normoxic controls. Cells were exposed to hypoxia for 0, 6, 12, 24, or 48 h to establish induction kinetics. SQOR mRNA and protein levels were measured across the time course by quantitative RT–PCR, using GAPDH as the internal control, and Western blotting, respectively; HIF-1α stabilization served as a positive control for hypoxia exposure. Downstream functional assays used the prespecified 24-h time point, which captured robust SQOR induction and the CoQH_2_/total CoQ shift while limiting confounding from prolonged hypoxia. For three-dimensional modeling, spheroids were generated in ultra-low-attachment plates and grown to a defined diameter before hypoxia exposure to better approximate the oxygen gradient in solid PDAC tumors. Mechanistic inference is therefore restricted to these two KRAS-mutant cellular backgrounds; phenotypic differences between them do not establish coverage of PDAC genetic or metabolic heterogeneity.

### 2.7 SQOR genetic manipulation constructs

Stable SQOR knockdown was achieved by lentiviral delivery of two independent, non-overlapping shRNA sequences targeting distinct regions of SQOR mRNA, followed by puromycin selection; scrambled shRNA served as the control. Knockdown was confirmed at both the mRNA and protein levels, and a reduction of at least 70% was required for inclusion in subsequent assays. Wild-type human SQOR was stably overexpressed from a lentiviral construct driven by a constitutive promoter, with empty-vector-transduced cells serving as controls. A catalytically dead SQOR mutant was generated by site-directed mutagenesis of the conserved catalytic cysteine required for sulfide oxidation. Western blotting confirmed expression comparable to that of wild-type SQOR, reducing the likelihood that differences in protein abundance accounted for the observed phenotypes. This comparison does not exclude effects of the mutation on protein folding or localization. This three-arm genetic system—SQOR knockdown, wild-type SQOR overexpression, and catalytically dead SQOR overexpression—was used consistently for all downstream functional readouts.

### 2.8 Electron donor validation

To identify the physiological electron donor supporting SQOR-mediated CoQ reduction, cells were treated with the slow-releasing H_2_S donor GYY4137 (100–400 µM) or sodium hydrosulfide (NaHS; 100–300 µM) and, separately, with sodium selenite as an H_2_Se-generating precursor across a dose range informed by previous redox-biology studies. The CoQH_2_ ratio and lipid ROS were then measured. To compare the endogenous contributions of the two pathways, the H_2_S-producing enzymes cystathionine β-synthase (CBS) and cystathionine γ-lyase (CSE) were inhibited pharmacologically with aminooxyacetic acid and propargylglycine, respectively, or silenced using siRNA. Selenoprotein/selenocompound metabolism was perturbed in parallel. Changes in the reduced CoQ fraction were compared across conditions to rank donor contributions empirically rather than assuming a single pathway.

### 2.9 CoQ redox-state quantification

Mitochondria were isolated from treated cells by differential centrifugation. Total CoQ and reduced CoQH_2_ were extracted using a hexane/ethanol-based lipid-extraction protocol under light-protected, argon-purged conditions to minimize artifactual auto-oxidation. CoQ and CoQH_2_ species were separated and quantified by HPLC with electrochemical detection. LC–MS/MS was performed in parallel on a subset of samples for cross-validation using authentic CoQ9 and CoQ10 standards. The reduced CoQ fraction was calculated as CoQH_2_/(CoQ + CoQH_2_) × 100% and was the primary CoQ redox readout across genetic and pharmacological conditions. Because other enzymes also reduce CoQ, this ratio alone is not an SQOR-specific target-engagement assay.

### 2.10 Lipid ROS detection

Whole-cell lipid peroxidation was measured by C11-BODIPY581/591 flow cytometry. Cells were loaded with the probe for 30 min at 37 °C, and the shift from reduced red fluorescence to oxidized green fluorescence was quantified by flow cytometry. Mitochondria-specific lipid peroxidation was measured in parallel with Mito-BODIPY or MitoPeDPP probes using confocal fluorescence microscopy and colocalization with MitoTracker. This approach distinguished mitochondrial from extramitochondrial oxidative burden at the organelle level, consistent with the mitochondria-centered study hypothesis.

### 2.11 Lipid peroxidation and lipidomics profiling

Malondialdehyde (MDA) content was measured using the thiobarbituric acid reactive substances (TBARS) colorimetric assay and normalized to protein concentration. Protein adducts of 4-HNE were detected by Western blotting with an anti-4-HNE antibody and quantified by ELISA for higher-throughput comparisons across treatment conditions. Untargeted and targeted lipidomic profiling was performed by LC–MS/MS using a high-resolution mass spectrometer coupled to reversed-phase chromatography. Analyses focused on oxidized phosphatidylethanolamine species containing arachidonic acid or adrenic acid (PE-AA and PE-AdA), which are directly implicated as lipid substrates in GPX4-regulated ferroptosis (Kagan et al., 2017). Four independent cultures were analyzed per condition. Pooled quality-control (QC) samples were injected at regular intervals throughout the analytical sequence. Features with a coefficient of variation >30% in QC samples or >20% missingness were excluded before differential analysis. The median QC coefficient of variation among retained lipid features was 12.8%. Figure 2A row Z-scores summarize within-lipid variation across the displayed samples; differential-abundance analysis used the criteria in Section 2.20. Oxidized PE annotations are treated as putative assignments: precursor mass alone does not establish the oxygenation site or distinguish isomers, which requires diagnostic fragmentation and chromatographic evidence (Criscuolo et al., 2022).

**FIGURE 2:**
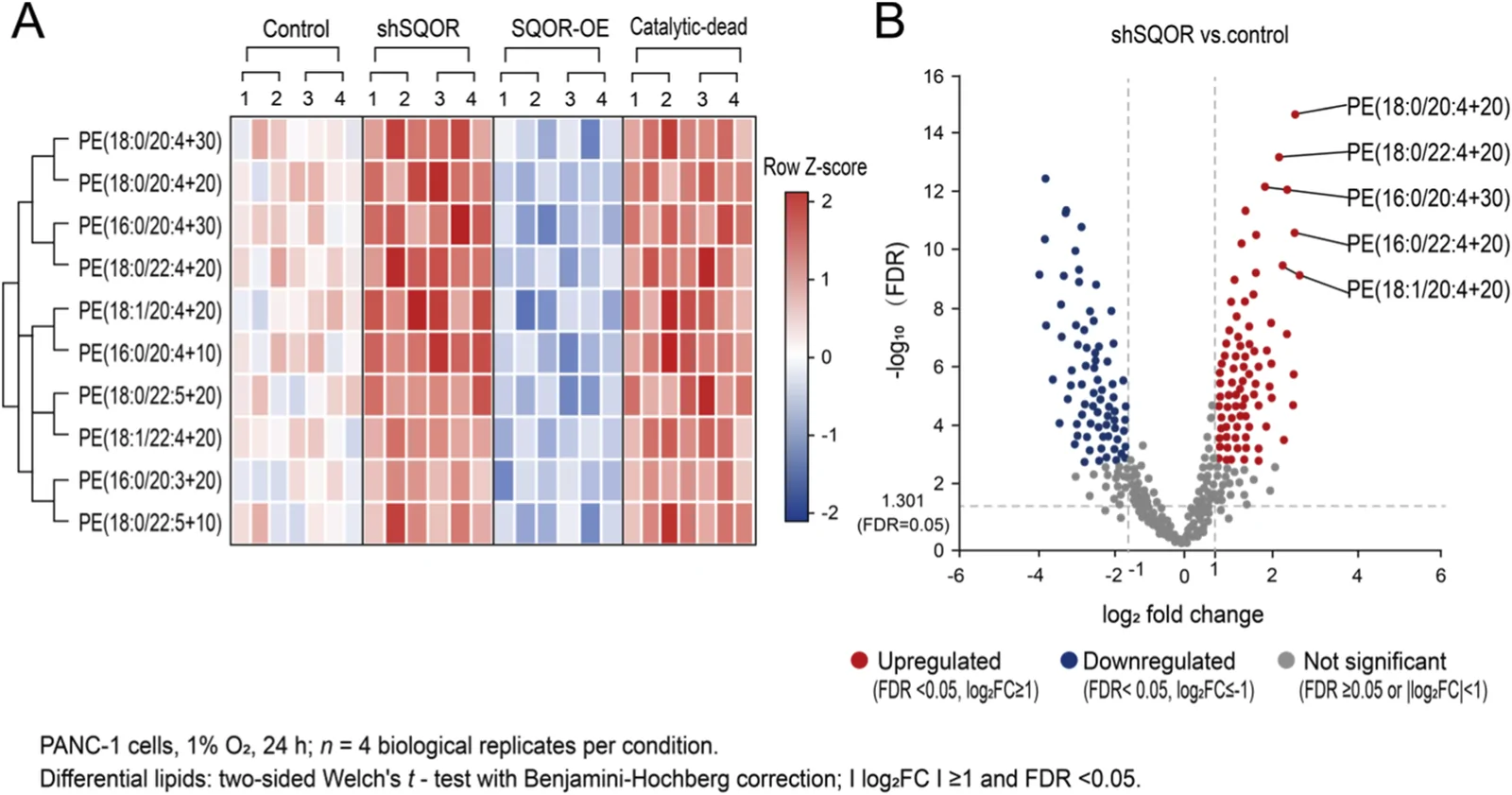
Oxidized PUFA-phospholipid profiling. **(A)** Row-standardized heatmap from four independent cultures per SQOR condition (n = 4). **(B)** Volcano plot for shSQOR versus control; differential species met FDR-adjusted q < 0.05 and |log_2_ fold change| ≥1. Lipid labels denote putative annotations, not confirmed oxidation-site or isomer assignments. Row Z-scores summarize relative patterns and are not absolute concentrations. For shSQOR versus control, raw P/BH-adjusted q values were: PE (18:0/20:4+2O), 0.00031/0.0042; PE (18:0/20:4+3O), 0.00074/0.0068; PE (16:0/20:4+2O), 0.0016/0.011; PE (18:0/22:4+2O), 0.0028/0.016; and PE (18:0/22:5+2O), 0.0061/0.028. The fold-change criterion was applied separately from the FDR criterion.

### 2.12 Ferroptosis sensitivity assays

Cells representing each SQOR genetic background—knockdown, wild-type overexpression, catalytically dead mutant expression, and the corresponding controls—were exposed for 24 h to escalating concentrations of the GPX4 inhibitor RSL3 or the system xc^−^ inhibitor erastin. Cell viability was measured by CCK-8 assay to generate dose–response curves and half-maximal inhibitory concentration (IC_50_) values. Clonogenic survival was assessed over 10–14 days to capture longer-term differences in sensitivity and thereby link CoQ and lipid-peroxidation status to functional ferroptosis susceptibility.

### 2.13 Epistasis combination treatments

To define the relationship between SQOR and established ferroptosis regulators, graded shSQOR perturbation was combined with RSL3 (GPX4 inhibition), iFSP1 (FSP1 inhibition), and either brequinar or BAY 2402234 (DHODH inhibition) in the combination matrices shown in Figure 3. Four independently repeated matrices were generated for each pairing. Technical wells within each matrix were averaged, and each independently repeated complete matrix constituted n = 1. In reciprocal rescue experiments, wild-type SQOR was overexpressed in GPX4-, FSP1-, or DHODH-deficient backgrounds (n = 5 independent biological experiments per background).

**FIGURE 3:**
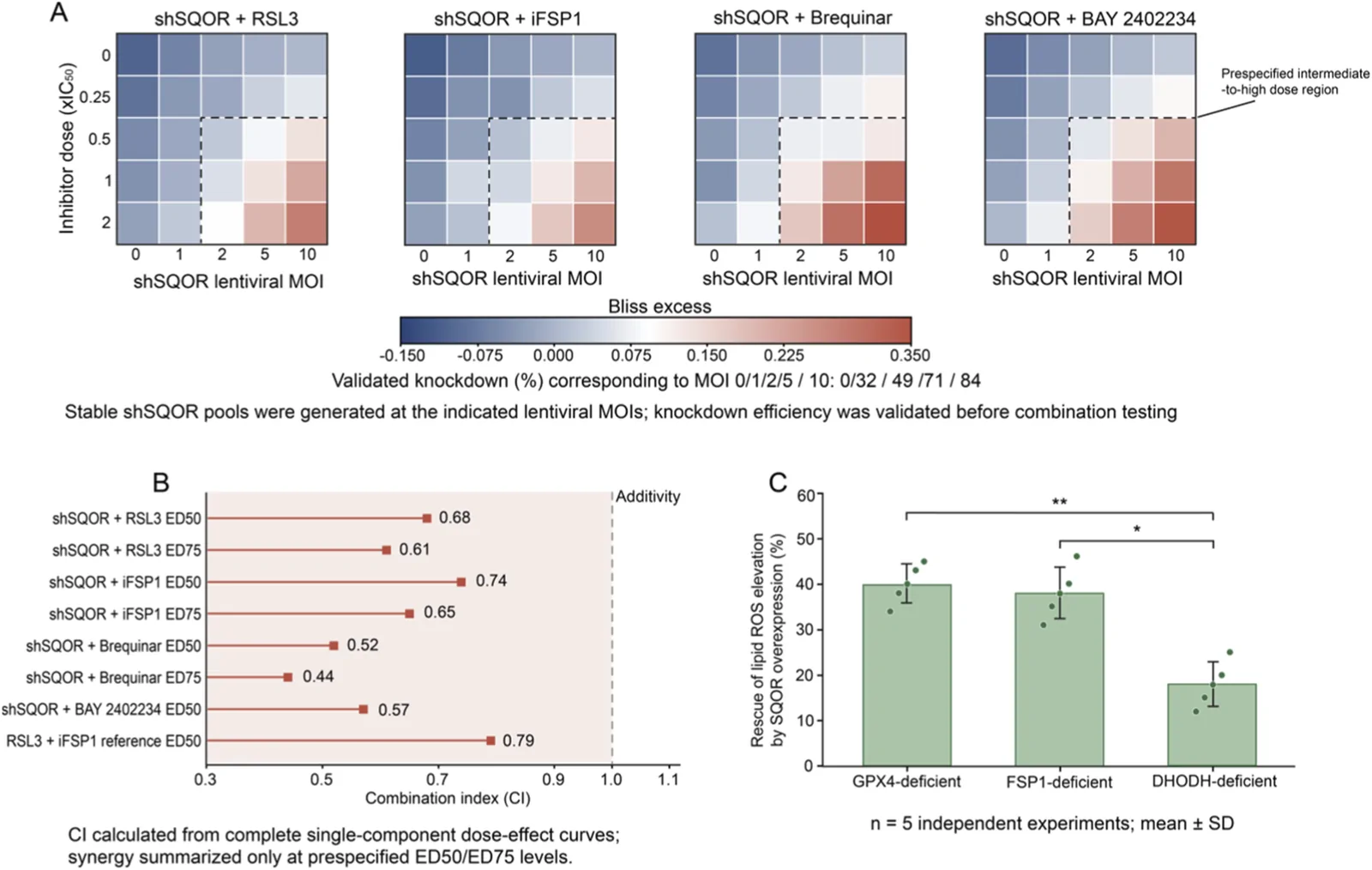
shSQOR-based pathway-epistasis analysis. **(A)** Bliss-excess matrices for shSQOR combined with RSL3, iFSP1, brequinar, or BAY 2402234 (n = 4 independent matrices per pair). **(B)** Chou-Talalay CI values from the matched dose-effect series. **(C)** Rescue of lipid-ROS elevation by SQOR overexpression in GPX4-, FSP1-, and DHODH-deficient backgrounds (n = 5 independent experiments; mean ± SD; one-way ANOVA with Tukey adjustment). For panel C, GPX4-deficient versus DHODH-deficient: raw P = 0.0017, Tukey-adjusted P = 0.0048; FSP1-deficient versus DHODH-deficient: raw P = 0.0039, adjusted P = 0.0106; GPX4-deficient versus FSP1-deficient: raw P = 0.594, adjusted P = 0.841 (ns).

### 2.14 Combination index analysis

Bliss excess was calculated at each shSQOR–inhibitor matrix point relative to the expected effect under independence. Chou–Talalay combination-index (CI) values were derived from the corresponding dose–effect series at ED50 and ED75 (Chou, 2010). CI < 1, CI = 1, and CI > 1 indicated synergism, additivity, and antagonism, respectively. Synergy was interpreted across dose regions rather than inferred from a single matrix point. Negative or near-zero Bliss values at low perturbation levels were retained and were not reclassified as synergistic.

### 2.15 Death mode discrimination

To assess the contribution of ferroptosis to viability loss under fixed-level shSQOR combination-treatment conditions, cells were co-treated with ferrostatin-1 (Fer-1; 1–2 µM), deferoxamine (DFO; 50–100 µM), or Z-VAD-FMK (20 µM) (Mishima et al., 2025). The no-rescue reference comprised the complete combination treatment plus the rescue-agent vehicle, rather than untreated vehicle. Rescue was calculated as (viability with rescue − viability with combination alone)/(100 − viability with combination alone) × 100. Fer-1 and DFO rescue fractions were reported separately as concordant indicators of ferroptosis and were not summed. Four independent experiments were performed in each cell line (total n = 8 biological observations per rescue condition), with cell line included as a blocking factor. CCK-8 is a metabolic viability readout. These values quantify recovery of assay viability and are not mutually exclusive proportions of cells assigned to distinct death mechanisms. Rescue specificity, incomplete protection, and metabolic effects on the viability assay prevent exact partitioning of ferroptotic, apoptotic, and residual death.

### 2.16 Resistant cell and organoid models

Gemcitabine-resistant PANC-1-GEM-R and MIA PaCa-2-GEM-R sublines were generated by continuous stepwise dose escalation over several months. Resistance was confirmed by a stable rightward shift in the IC_50_ after a drug-free passage interval. Eight PDO lines were independently established from eight patients using established pancreatic organoid methods; each patient contributed one analytical PDO line (Boilève et al., 2024; Boj et al., 2015). SQOR abundance was measured in the corresponding parent-tumor specimen before drug testing, and a prespecified median split classified four lines as SQOR-high and four as SQOR-low. Technical organoid wells were averaged within each PDO line before analysis (Takeuchi et al., 2025; Tiriac et al., 2018). This within-series median split is descriptive and is distinct from the outcome-optimized clinical TMA cutoff; neither threshold constitutes a validated predictor of treatment benefit.

### 2.17 Drug sensitivity assays in resistant models

SQOR mRNA abundance, protein expression, and catalytic activity were compared between parental and resistant cell lines, together with baseline CoQH_2_ ratios and lipid-peroxidation markers. Gemcitabine IC_50_ values were estimated in parental and resistant cells with or without the SQOR inhibitor and with or without a ferroptosis inducer. Four independent CCK-8 dose–response experiments were performed per condition, and technical wells were averaged before four-parameter nonlinear regression. Clonogenic assays provided an orthogonal measure of long-term sensitivity. The compound designated “SQOR inhibitor” was evaluated as a putative SQOR-directed agent in exploratory combination-response experiments.

### 2.18 *In vivo* models and treatment intervention

Subcutaneous xenografts were generated from gemcitabine-resistant PDAC cells. Once tumors reached the prespecified baseline volume, mice were randomized to vehicle, gemcitabine, the SQOR inhibitor, or their combination. The tumor-growth/terminal-weight cohort included n = 6 mice per group. Blinded assessors measured tumor volume at prespecified intervals, and tumors were weighed at the terminal assessment on day 28. A separate survival cohort included n = 7 mice per group and was followed until the prespecified humane endpoint. No animal contributed observations to both cohorts. Tumor growth, terminal weight, and survival were efficacy endpoints. These endpoints alone do not demonstrate SQOR target engagement or ferroptotic tumor-cell death *in vivo*.

### 2.19 Analysis sets, experimental units, and replication

The patient was the independent unit in clinical analyses (N = 102). Cell-based hypoxia and SQOR-manipulation analyses comprised five independent experiments per cell line; when both cell lines were displayed together, the total biological n was 10, and cell line was modeled as a blocking factor. Electron-donor experiments used n = 5 independent PANC-1 cultures per condition, and lipidomic analyses used n = 4 independent cultures per condition. The shSQOR combination analyses comprised n = 4 independently repeated matrices, reciprocal-rescue experiments comprised n = 5 independent experiments per background, and death-mode rescue assays comprised n = 4 experiments per cell line (total n = 8). Resistant-cell dose–response analyses used n = 4 independent experiments per condition. The PDO analysis set comprised eight patient-derived lines (four SQOR-high and four SQOR-low). Xenograft growth/weight and survival cohorts included n = 6 and n = 7 mice per group, respectively. Technical wells, microscopic fields, duplicate cores, and repeated measurements within the same mouse were not counted as independent replicates.

### 2.20 Statistical analysis

Analyses were performed using SPSS 26.0, GraphPad Prism 8.0, and R 4.2.0. Continuous data are presented as the mean ± SD unless otherwise stated; Kaplan–Meier curves display survival probabilities and censoring marks. Two-group comparisons used two-sided Welch t-tests. Three or more independent groups were compared by one-way ANOVA with Tukey-adjusted pairwise tests. Factorial cell experiments were analyzed by two-way ANOVA or linear mixed-effects models, with experiment and cell line treated as blocking/random effects, followed by Tukey-adjusted pairwise comparisons for the SQOR-manipulation experiments (Figure 4) or Šidák-adjusted planned contrasts for the death-mode rescue experiments (Figure 5). PDO data were analyzed using a mixed-effects model with treatment, SQOR subgroup, and their interaction as fixed effects and PDO line as a random intercept. Longitudinal tumor volumes were analyzed using a mixed-effects model containing treatment, time, and the treatment × time interaction, with mouse as a random intercept; day-28 contrasts were Tukey-adjusted. Terminal tumor weights were analyzed by one-way ANOVA with Tukey adjustment, and survival was analyzed using log-rank tests with Holm adjustment. Four-parameter logistic models were used to estimate IC_50_ values; curves were compared using an extra-sum-of-squares F test with Holm–Šidák correction. Lipidomic comparisons used two-sided Welch tests with Benjamini–Hochberg correction; significance required q < 0.05 and |log_2_ fold change| ≥1. Two-sided tests were used where applicable. Exact biological n values are reported in Section 2.19 and the figure legends. Significance symbols denote ns, adjusted P ≥ 0.05; *, adjusted P < 0.05; **, adjusted P < 0.01; and ***, adjusted P < 0.001. For the six-marker correlation matrix and lipidomic analyses, q denotes the Benjamini–Hochberg FDR-adjusted P value. Other multiplicity-adjusted P values are identified by their adjustment method. Thresholds used to define significance are distinct from the numerical P or q values of individual tests. Table 3 reports numerical raw P values and BH-adjusted q values for all 15 marker pairs, including nonsignificant results; scientific notation is used for very small values. The clinical response contingency analysis used an uncorrected Pearson chi-square test with one degree of freedom. A two-sided Fisher exact test was performed for the same aggregate 2 × 2 table by summing fixed-margin table probabilities no greater than that of the observed table. An unadjusted response odds ratio for SQOR-high versus SQOR-low was calculated, with a 95% Wald confidence interval on the log-odds scale. The contingency-table analyses assessed the unadjusted association with the mixed response endpoint. Table 4 reports Dunnett-adjusted comparisons against the hypoxia control.

**FIGURE 4:**
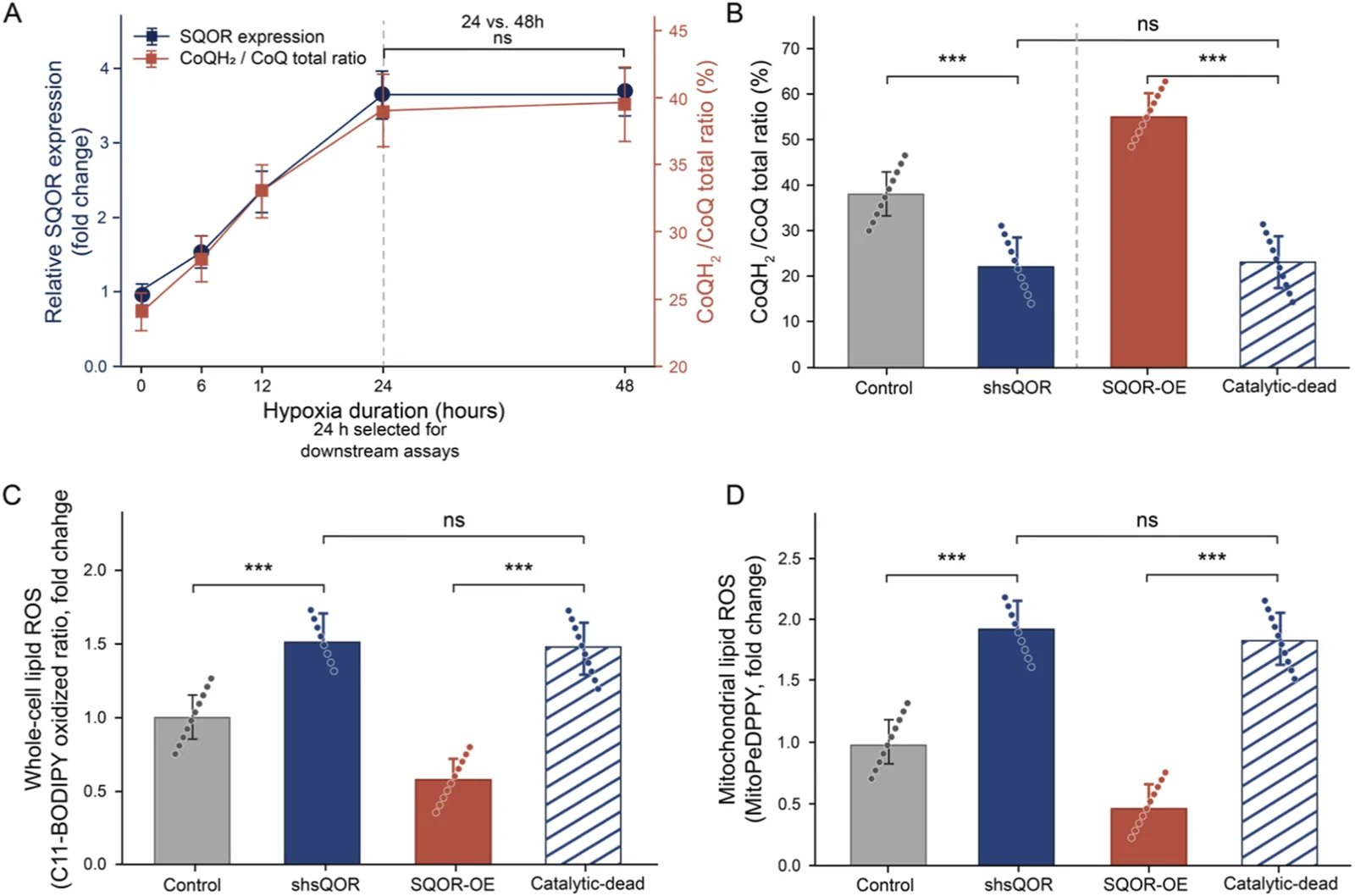
Hypoxia time course and catalysis-dependent SQOR effects. **(A)** SQOR expression and CoQH_2_/total CoQ ratio across 0–48 h of hypoxia. **(B–D)** CoQH_2_/total CoQ ratio, whole-cell lipid ROS, and mitochondrial lipid ROS across control, shSQOR, SQOR-OE, and catalytic-dead conditions. Data are mean ± SD from five independent experiments per cell line (total n = 10 per group when pooled); cell line was treated as a blocking factor and Tukey-adjusted contrasts were used. ns, adjusted P ≥ 0.05; *, adjusted P < 0.05; **, adjusted P < 0.01; ***, adjusted P < 0.001. In the comparison order control versus shSQOR, control versus SQOR-OE, control versus catalytic-dead, shSQOR versus SQOR-OE, SQOR-OE versus catalytic-dead, and shSQOR versus catalytic-dead, Tukey-adjusted P values were: **(B)** 0.0007, <0.0001, 0.0011, <0.0001, <0.0001, and 0.781; **(C)** 0.0004, 0.0005, 0.0007, <0.0001, <0.0001, and 0.663; **(D)** < 0.0001, 0.0003, <0.0001, <0.0001, <0.0001, and 0.512. Raw P values for control versus shSQOR, control versus SQOR-OE, control versus catalytic-dead, and shSQOR versus catalytic-dead in **(B)** were 0.00018, <0.000001, 0.00031, and 0.438, respectively. In **(C)**, raw P values for control versus shSQOR, control versus SQOR-OE, and shSQOR versus catalytic-dead were 0.00009, 0.00012, and 0.317, respectively. In **(D)**, raw P = 0.214 for shSQOR versus catalytic-dead.

**FIGURE 5:**
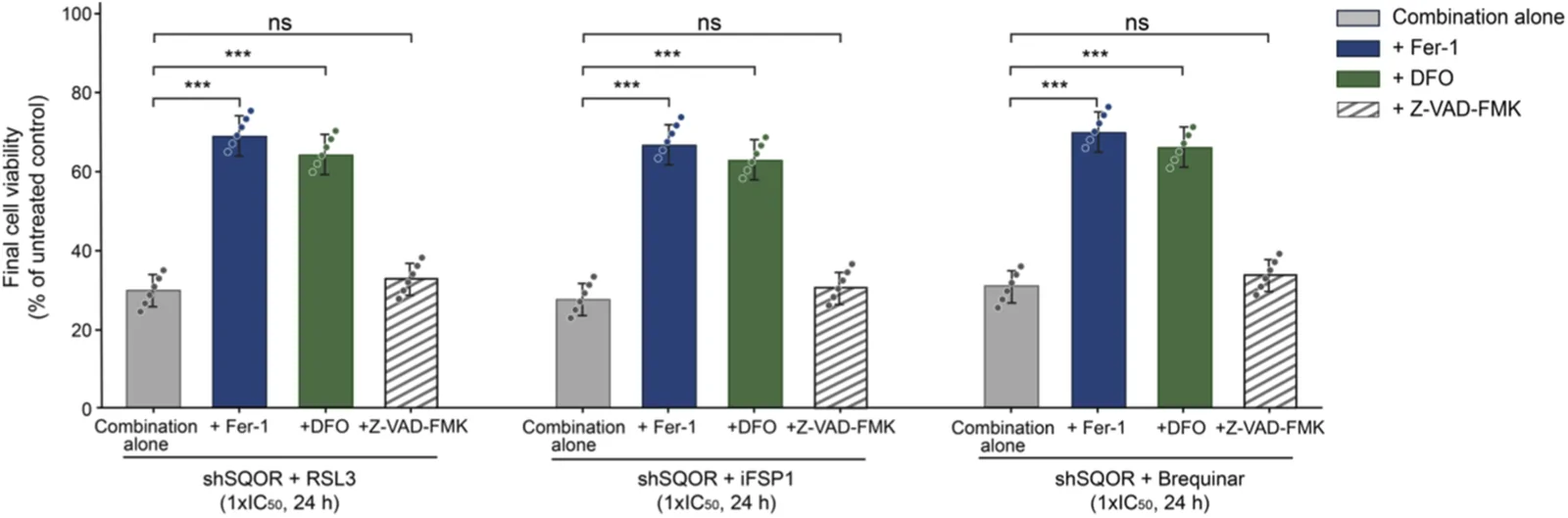
Viability rescue under shSQOR combination treatments. The figure shows final cell viability after Fer-1, DFO, or Z-VAD-FMK co-treatment under three fixed shSQOR combination conditions. The no-rescue reference is combination treatment plus rescue-agent vehicle. Data are mean ± SD from four independent experiments per cell line (total n = 8 per condition), analyzed using a two-way model with cell line as a blocking factor and Šidák-adjusted planned contrasts. ns, adjusted P ≥ 0.05; *, adjusted P < 0.05; **, adjusted P < 0.01; ***, adjusted P < 0.001. Rescue supports mechanistic interpretation but does not partition death into mutually exclusive fractions.

**TABLE 3:** Pairwise correlations among protein markers in the PDAC cohort.

Marker pair	ρ	Raw P	BH-adjusted q
CAIX/HIF-1α	0.568	4.60228 × 10^−10^	6.90343 × 10^−9^
CAIX/SQOR	0.473	5.23393 × 10^−7^	3.92544 × 10^−6^
CAIX/4-HNE	−0.291	0.00297318	0.00891955
CAIX/GPX4	−0.142	0.153851	0.192313
CAIX/FSP1	−0.098	0.328257	0.351704
HIF-1α/SQOR	0.438	4.21016 × 10^−6^	1.57881 × 10^−5^
HIF-1α/4-HNE	−0.201	0.0424338	0.0795635
HIF-1α/GPX4	−0.210	0.0340155	0.0795635
HIF-1α/FSP1	−0.153	0.123938	0.169007
SQOR/4-HNE	−0.465	8.27962 × 10^−7^	4.13981 × 10^−6^
SQOR/GPX4	−0.175	0.0791828	0.118774
SQOR/FSP1	−0.076	0.447583	0.447583
4-HNE/GPX4	0.205	0.0387301	0.0795635
4-HNE/FSP1	0.118	0.236464	0.272843
GPX4/FSP1	0.195	0.0497586	0.082931

All 15 unique pairs were analyzed using two-sided Spearman rank correlations (n = 102 patients after averaging duplicate TMA cores). P denotes the unadjusted value; q denotes the Benjamini–Hochberg FDR-adjusted value across all 15 tests. CAIX and HIF-1α are tissue hypoxia markers, and 4-HNE is a lipid-peroxidation marker. These associations do not establish gene-transcript coexpression, molecular interactions, or causal mediation.

**TABLE 4:** CoQH_2_/total CoQ ratio and lipid ROS under electron-donor manipulation (PANC-1, 24 h hypoxia; n = 5 independent cultures per condition).

Condition	Reduced CoQ fraction (%)	Whole-cell lipid ROS (fold vs. hypoxia control)	Raw P/Dunnett-adjusted P (reduced CoQ fraction vs. hypoxia control)
Hypoxia control	36.7 ± 2.2	0.99 ± 0.05	—
+ GYY4137 (200 µM)	47.5 ± 7.5	0.62 ± 0.13	0.0011/0.0030
+ NaHS (200 µM)	50.9 ± 7.0	0.70 ± 0.09	0.0046/0.0120
+ Sodium selenite (5 µM)	38.9 ± 4.9	1.04 ± 0.20	0.0914/0.1870
CBS inhibition (AOAA)	24.4 ± 5.2	1.57 ± 0.23	0.0003/0.0010
CSE knockdown (siCSE)	28.5 ± 5.1	1.41 ± 0.09	0.0014/0.0040
Selenium metabolism perturbation	39.2 ± 3.2	1.10 ± 0.13	0.1817/0.3410

Values are mean ± SD from five independent PANC-1 cultures. Each P-value entry gives the raw P value followed by the Dunnett-adjusted P value for the reduced CoQ fraction compared with the common hypoxia control. The six comparisons include the nonsignificant sodium-selenite and selenium-metabolism-perturbation results. Lipid-ROS values are reported as the matched mechanistic readout.

## 3 Results

### 3.1 SQOR protein abundance is associated with hypoxia-marker expression and lower 4-HNE staining

In the patient-level clinical analysis set (n = 102), duplicate TMA cores were averaged before correlation analysis. SQOR H-score showed moderate positive associations with CAIX (Spearman ρ = 0.473, raw P = 5.23393 × 10^−7^, BH-adjusted q = 3.92544 × 10^−6^) and HIF-1α (ρ = 0.438, raw P = 4.21016 × 10^−6^, q = 1.57881 × 10^−5^), and a moderate inverse association with 4-HNE (ρ = −0.465, raw P = 8.27962 × 10^−7^, q = 4.13981 × 10^−6^; Table 3). These correlations were moderate in magnitude. CAIX and HIF-1α were moderately correlated (ρ = 0.568, raw P = 4.60228 × 10^−10^, q = 6.90343 × 10^−9^). The inverse CAIX–4-HNE association was weaker but remained significant after correction across the 15 pairwise tests (ρ = −0.291, raw P = 0.00297318, q = 0.00891955), whereas the HIF-1α–4-HNE association did not (ρ = −0.201, raw P = 0.0424338, q = 0.0795635). SQOR showed weak, nonsignificant associations with GPX4 (ρ = −0.175, raw P = 0.0791828, q = 0.118774) and FSP1 (ρ = −0.076, raw P = 0.447583, q = 0.447583). All 15 pairwise results, including nonsignificant tests, are reported in Table 3. The X-tile-derived cutoff classified 47 patients as SQOR-high and 55 as SQOR-low for all response and survival analyses. These cross-sectional protein-marker associations support an association of higher SQOR abundance with hypoxia-marker expression and lower 4-HNE staining. They do not establish a consistent inverse association between both hypoxia markers and 4-HNE after FDR correction, nor do they demonstrate that hypoxia causes lower lipid peroxidation, that SQOR mediates such an effect in patients, or that lower 4-HNE establishes reduced ferroptotic cell death.

### 3.2 High SQOR expression is associated with lower chemotherapy response and shorter survival

Under the exploratory mixed-endpoint classification, 14 of 47 SQOR-high patients (29.8%) and 30 of 55 SQOR-low patients (54.5%) were classified as responders (Pearson χ^2^ (1) = 6.333, P = 0.011850). The unadjusted response odds ratio for SQOR-high versus SQOR-low was 0.354 (95% Wald CI, 0.156–0.803); the two-sided Fisher exact P value was 0.016067. Both tests support an association within the mixed classification, without resolving differences between radiological and biochemical assessment. During follow-up, 75 deaths occurred among the 102 patients, with 27 patients censored: 40 of 47 SQOR-high patients and 35 of 55 SQOR-low patients died. Overall survival curves separated early and remained distinct, with a median overall survival of 14.2 months in the SQOR-high group and 23.8 months in the SQOR-low group (log-rank P = 2.68 × 10^−4^; Figure 6A). The accompanying risk table documents the progressive reduction in patients remaining under observation and provides context for the late portions of the curves. Progression-free survival was also shorter in the SQOR-high group (log-rank P = 0.00199; Figure 6B), indicating that the adverse association extended to disease control.

**FIGURE 6:**
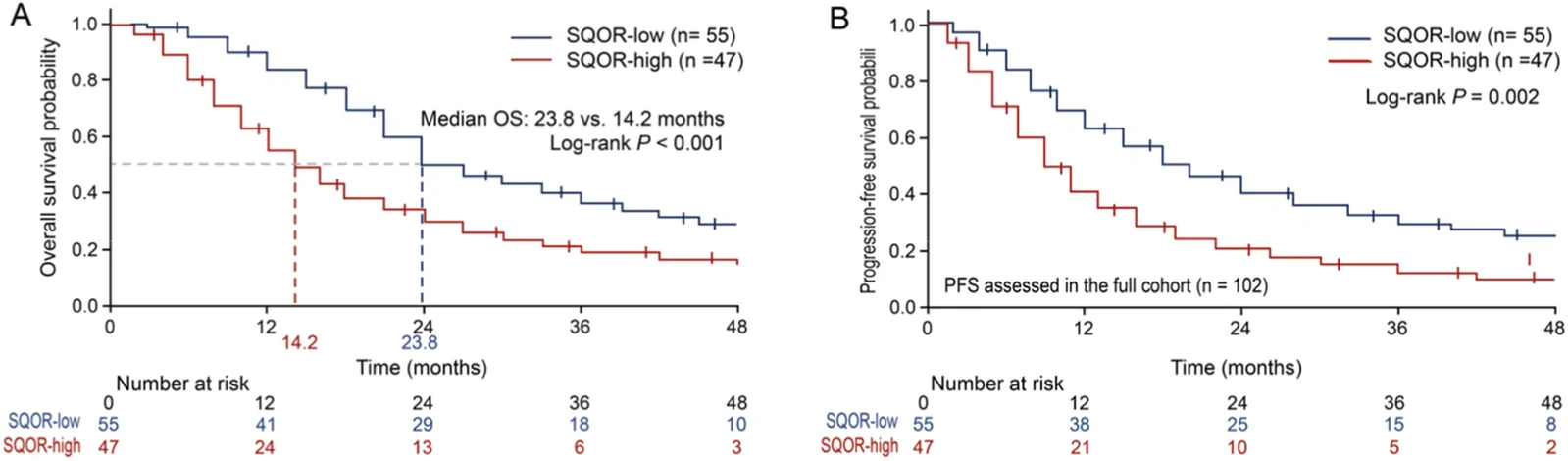
Survival and prognostic analyses stratified by SQOR expression. **(A)** Overall survival and **(B)** progression-free survival in SQOR-low (n = 55) and SQOR-high (n = 47) patients, compared by two-sided log-rank tests. Cox model estimates are presented in Table 2. Numerical log-rank P values were 2.68 × 10^−4^ for overall survival and 0.00199 for progression-free survival.

Both Cox models included all 102 patients and 75 observed deaths, corresponding to 12.5 and approximately 9.4 events per fitted parameter in Models 1 and 2, respectively. In Cox Model 1, high SQOR expression was associated with an increased mortality hazard after adjustment for the included covariates (HR, 2.14; 95% CI, 1.32–3.47; P = 0.002). Advanced TNM stage (HR, 2.63; 95% CI, 1.58–4.38; P = 1.99 × 10^−4^) and lymph-node metastasis (HR, 1.79; 95% CI, 1.08–2.97; P = 0.024) were also associated with higher mortality within Model 1. Additional adjustment for GPX4 and FSP1 in Model 2 attenuated the SQOR estimate, which remained significant (HR 1.87, 95% CI 1.11–3.15; P = 0.019), whereas neither GPX4 nor FSP1 was individually significant (Table 2). Thus, the prognostic association of SQOR partially overlapped with, but was not subsumed by, these canonical ferroptosis-defense proteins. The model-specific estimates in Table 2 derive from two separate prespecified Cox models and should not be interpreted as simultaneous coefficients from a single model. Schoenfeld residual tests did not provide evidence against the proportional-hazards assumption: global P values were 0.64 for Model 1 and 0.61 for Model 2, and SQOR-specific P values were 0.71 and 0.68, respectively. These estimates remain exploratory because same-cohort cut-point selection, unquantified model optimism, and residual clinical confounding limit their interpretation.

### 3.3 Hypoxia induces SQOR-driven, catalysis-dependent suppression of lipid peroxidation

In both PANC-1 and MIA PaCa-2 cells, SQOR expression increased during hypoxia, was robustly elevated by 24 h, and rose only modestly thereafter through 48 h (Figure 4A). The 24-h time point was prespecified for downstream experiments because it produced a strong shift in the CoQH_2_/total CoQ ratio while limiting potential confounding from prolonged hypoxia; it was not considered the absolute maximum of SQOR expression. At 24 h, the CoQH_2_/total CoQ ratio was approximately 1.6-fold higher than the normoxic baseline.

Across five independent experiments per cell line, SQOR knockdown reduced the CoQH_2_/total CoQ ratio and increased both whole-cell and mitochondrial lipid ROS (Figures 4B–D). Wild-type SQOR overexpression produced the reciprocal pattern, whereas the catalytically dead mutant resembled SQOR knockdown and differed clearly from wild-type overexpression. For mitochondrial lipid ROS, the largest separation was between wild-type overexpression and the two loss-of-function conditions. The consistent ordering across CoQ redox status and both compartment-resolved lipid-oxidation readouts indicates that the protective phenotype depended on SQOR catalytic activity rather than protein abundance alone.

### 3.4 Electron-donor dependence of SQOR-mediated CoQ reduction

Supplementation with the H_2_S donors GYY4137 and NaHS increased the reduced CoQ fraction in a dose-dependent manner and reduced lipid ROS, whereas sodium selenite had a comparatively modest effect (Table 4). Pharmacological or genetic inhibition of the H_2_S-producing enzymes CBS and CSE substantially blunted the hypoxia-associated increase in reduced CoQ. By contrast, perturbation of selenium metabolism produced a smaller, nonsignificant change. Under the tested conditions, perturbation of H_2_S-generating pathways had a larger effect on the CoQ redox state than perturbation of selenium metabolism. These findings support, but do not establish, a predominant contribution from H_2_S; a selenium-dependent contribution cannot be excluded.

### 3.5 Lipidomic profiling reveals suppression of oxidized PUFA phospholipids

Targeted lipidomic profiling showed condition-associated changes in features annotated as oxidized phosphatidylethanolamines. SQOR knockdown and the catalytically dead mutant had higher relative signals assigned to oxidized PE-AA and PE-AdA than control, whereas wild-type SQOR overexpression had lower signals (Figure 2A). Features annotated as doubly and triply oxygenated PE-AA were among those altered. This pattern is consistent with increased phospholipid oxidation after SQOR loss, but unambiguous molecular and isomer assignments require analyte-specific MS/MS evidence.

The row-standardized heatmap showed a reproducible condition-dependent signature across four independent cultures per group: shSQOR and catalytically dead SQOR had higher standardized abundances of multiple oxidized PE species, whereas wild-type SQOR overexpression produced a broadly lower-abundance profile (Figure 2A). Because the values are row Z-scores, each lipid species should be compared across conditions rather than interpreted as an absolute comparison among species. In the shSQOR-versus-control analysis, significantly increased species clustered on the positive log_2_-fold-change side and included several oxidized PUFA-containing PE species, whereas fewer qualifying species were decreased (Figure 2B). Applying both FDR q < 0.05 and an absolute log_2_-fold-change threshold of at least 1 restricted interpretation to changes that were statistically supported and of meaningful magnitude. Together, these relative-abundance data support an association between SQOR loss and an oxidized-phospholipid signature. They do not independently verify specific death-executing lipid structures or exclude *ex vivo* oxidation artifacts.

### 3.6 The SQOR-CoQ axis makes a nonredundant contribution to ferroptosis control

Positive Bliss-excess values for shSQOR combined with RSL3, iFSP1, brequinar, or BAY 2402234 were concentrated in the prespecified intermediate-to-high perturbation regions (Figure 3A). Antagonistic or approximately additive responses remained at lower perturbation levels, indicating that synergy was dose-region dependent rather than uniform across each matrix. Chou–Talalay analysis of matched dose–effect series confirmed this pattern (Figure 3B; Table 5). CI values were below 1 for shSQOR plus RSL3 (0.68 at ED50 and 0.61 at ED75), shSQOR plus iFSP1 (0.74 and 0.65), shSQOR plus brequinar (0.52 and 0.44), and shSQOR plus BAY 2402234 (0.57 at ED50). The RSL3 plus iFSP1 reference pair showed weaker synergism (CI = 0.79 at ED50). The lowest CI values occurred with DHODH inhibition.

**TABLE 5:** Chou-Talalay combination-index values for shSQOR combined with pathway inhibitors (n = 4 independent matrices per pair).

Combination	Effect level	CI value	Interpretation
shSQOR + RSL3	ED50	0.68	Synergism
shSQOR + RSL3	ED75	0.61	Synergism
shSQOR + iFSP1	ED50	0.74	Synergism
shSQOR + iFSP1	ED75	0.65	Synergism
shSQOR + Brequinar	ED50	0.52	Synergism
shSQOR + Brequinar	ED75	0.44	Synergism
shSQOR + BAY 2402234	ED50	0.57	Synergism
RSL3 + iFSP1 (reference pair)	ED50	0.79	Synergism

CI values were derived from four independently repeated shSQOR-inhibitor matrices using the matched dose-effect series. CI < 1 indicates synergism, CI = 1 additivity, and CI > 1 antagonism.

Wild-type SQOR overexpression rescued approximately 40% and 38% of the lipid-ROS elevation in GPX4‐ and FSP1‐deficient cells, respectively, but only approximately 18% in the DHODH-deficient background (Figure 3C). The incomplete rescue in all three backgrounds supports a nonredundant contribution from SQOR. The smaller rescue in DHODH-deficient cells is consistent with convergence on mitochondrial CoQ redox control, but the combination data do not uniquely establish pathway proximity. Brequinar can also inhibit SQOR in biochemical assays (IC_50_ approximately 3.6 µM; Jackson et al., 2022), so its contribution requires exposure-specific interpretation alongside the BAY 2402234 and genetic results.

### 3.7 Rescue assays support a substantial ferroptotic contribution

Under fixed-level shSQOR combination-treatment conditions, the no-rescue reference retained approximately 27%–31% viability. Fer-1 increased final viability to approximately 67%–70%, and DFO increased it to approximately 62%–66%; both interventions provided significant rescue relative to the no-rescue reference (Figure 5). In contrast, Z-VAD-FMK produced only a small increase, to approximately 30%–34%, and did not differ significantly from combination treatment alone. Rescue fractions were normalized to the combination-associated viability deficit relative to untreated control; Fer-1 and DFO were analyzed separately rather than summed.

The greater rescue by Fer-1 and DFO than by Z-VAD-FMK supports a substantial ferroptotic contribution under these conditions. Together with the genetic SQOR perturbations and lipid-peroxidation readouts, the pattern supports a substantial ferroptotic contribution to the observed viability loss. However, pharmacological rescue does not quantify mutually exclusive percentages of ferroptotic and apoptotic death, and incomplete rescue leaves other mechanisms unresolved.

### 3.8 Putative SQOR-directed treatment enhances gemcitabine activity in resistant models

Gemcitabine-resistant PANC-1-GEM-R and MIA PaCa-2-GEM-R cells showed higher SQOR abundance and activity, a higher baseline CoQH_2_/total CoQ ratio, and lower baseline lipid ROS than their parental counterparts. In PANC-1-GEM-R cells, SQOR protein was approximately 2.8-fold higher than in parental cells, the CoQH_2_/total CoQ ratio increased from approximately 27% to 49%, and baseline lipid ROS fell to roughly half of the parental level (Figure 7A). This coordinated pattern is consistent with constitutive engagement of the SQOR–CoQ antioxidant axis during acquired resistance.

**FIGURE 7:**
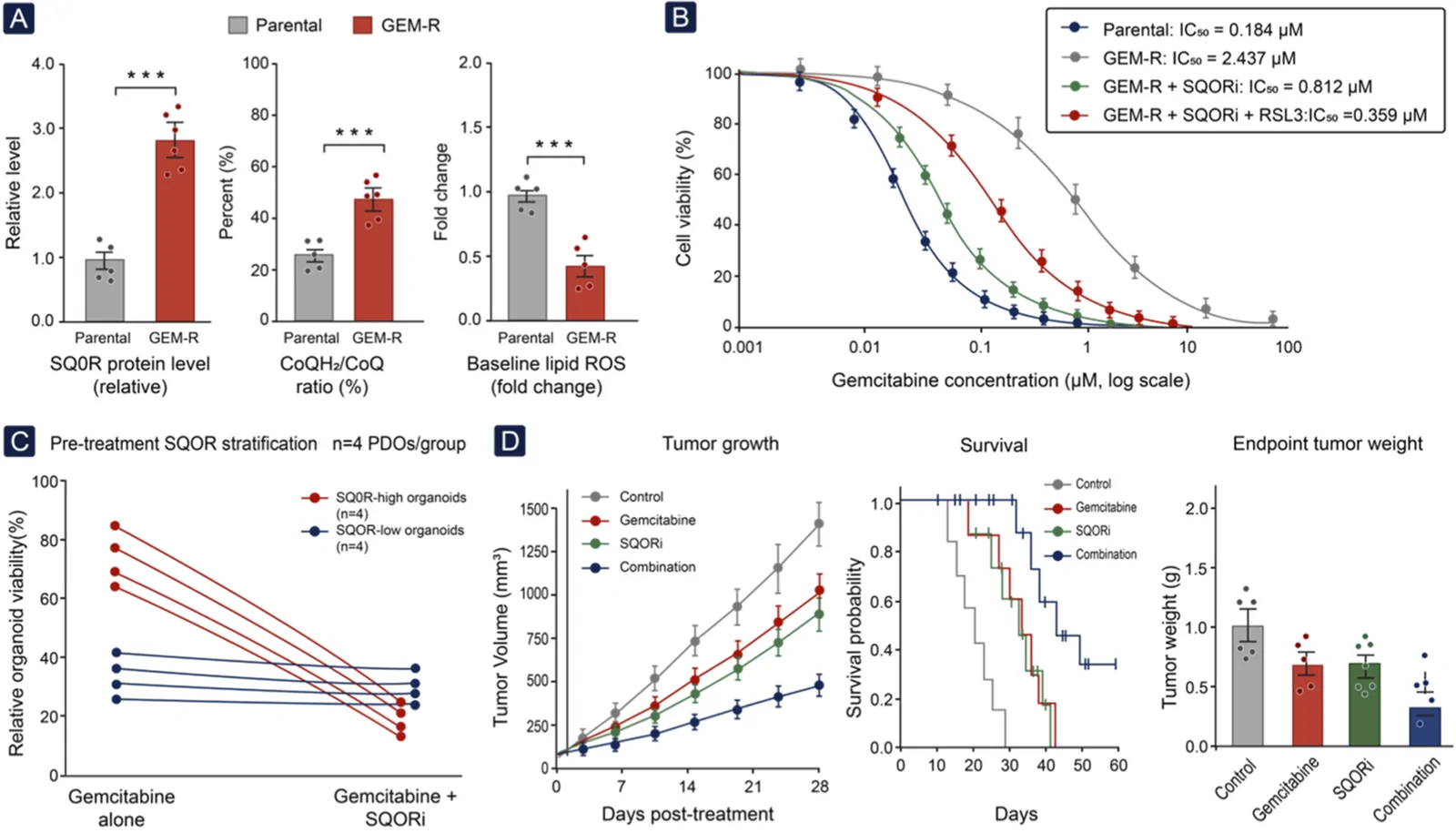
Characterization of gemcitabine-resistant PDAC models and response to SQOR-targeted combination treatment. **(A)** Relative SQOR protein expression, CoQH_2_/total CoQ ratio, and baseline lipid ROS in parental and PANC-1-GEM-R cells. **(B)** Gemcitabine dose–response curves in parental, GEM-R, GEM-R plus SQOR inhibitor, and GEM-R plus SQOR inhibitor and RSL3 conditions; corresponding IC_50_ values are indicated. **(C)** Paired PDO viability following gemcitabine alone or gemcitabine plus SQOR inhibitor, stratified according to pretreatment SQOR status (n = 4 PDOs per group). **(D)** Tumor growth and terminal tumor weight in one cohort (n = 6 mice per group), and survival in a separate cohort (n = 7 mice per group), under vehicle, gemcitabine, SQOR inhibitor, and combination treatment. SQORi denotes the putative SQOR-directed compound. (C) The exploratory treatment × SQOR-subgroup interaction had P = 0.0036. Panel D, combination versus gemcitabine alone: day-28 volume, raw P = 0.0013 and Tukey-adjusted P = 0.0040; terminal weight, raw P = 0.0019 and Tukey-adjusted P = 0.0060; survival, raw log-rank P = 0.0071 and Holm-adjusted P = 0.0210. Panel D shows efficacy endpoints and does not establish *in vivo* target engagement or ferroptosis.

The gemcitabine IC_50_ increased from 0.184 µM in parental PANC-1 cells to 2.437 µM in PANC-1-GEM-R cells, a 13.2-fold shift (Figure 7B; Table 6). Treatment with the putative SQOR inhibitor lowered the resistant-cell IC_50_ to 0.812 µM, and adding RSL3 reduced it further to 0.359 µM. These values remained 4.4-fold and 2.0-fold above the parental IC_50_, respectively. The putative SQOR inhibitor likewise reduced the gemcitabine IC_50_ in MIA PaCa-2-GEM-R cells, and the addition of erastin produced a further reduction (Table 6). Following putative SQOR-inhibitor treatment alone, MIA PaCa-2-GEM-R cells retained 4.7-fold resistance relative to the parental line. Thus, the putative SQOR-inhibitor treatment partially resensitized both resistant models rather than fully reversing resistance, with a larger effect when combined with ferroptosis induction.

**TABLE 6:** Gemcitabine IC_50_ values across parental, resistant, and SQOR-inhibitor treatment conditions (n = 4 independent experiments per condition).

Cell model/condition	Gemcitabine IC50 (µM)	Fold change vs parental	Raw P/Holm–Šidák-adjusted P	Comparator
PANC-1 parental	0.184 ± 0.021	1	—	—
PANC-1-GEM-R	2.437 ± 0.318	13.2	—	—
PANC-1-GEM-R + SQOR inhibitor	0.812 ± 0.096	4.4	1.4 × 10^−5^/5.6 × 10^−5^	GEM-R
PANC-1-GEM-R + SQOR inhibitor + RSL3	0.359 ± 0.058	2.0	<1 × 10^−6^/4 × 10^−6^	GEM-R
MIA PaCa-2 parental	0.221 ± 0.027	1	—	—
MIA PaCa-2-GEM-R	3.108 ± 0.402	14.1	—	—
MIA PaCa-2-GEM-R + SQOR inhibitor	1.045 ± 0.134	4.7	2.1 × 10^−5^/6.3 × 10^−5^	GEM-R
MIA PaCa-2-GEM-R + SQOR inhibitor + Erastin	0.427 ± 0.071	1.9	<1 × 10^−6^/3 × 10^−6^	GEM-R

Values are mean ± SD from four independent dose‐response experiments. Technical wells were averaged within each experiment. Curves were fitted by four-parameter nonlinear regression; the comparison procedure was an extra-sum-of-squares F test with Holm–Šidák correction. For inhibitor-treated resistant cells, the comparator was the corresponding untreated GEM‐R condition. Each P-value entry gives the raw P followed by the Holm–Šidák-adjusted P. Dashes indicate that no statistical comparison is presented. Fold changes are descriptive ratios of the displayed mean IC_50_ to the corresponding parental mean; they do not identify the comparator used for a statistical test.

Across eight independent PDO lines classified by parent-tumor SQOR abundance before drug testing, adding the putative SQOR-directed compound to gemcitabine reduced viability in all four SQOR-high lines by 46–52 percentage points (mean paired change, −49.0 percentage points). Changes in the four SQOR-low lines ranged from −11 to +3 percentage points (mean paired change, −4.75 percentage points; Figure 7C). The unadjusted difference in mean paired changes, high minus low, was −44.25 percentage points. An exploratory mixed-effects analysis gave P = 0.0036 for the treatment × SQOR-subgroup interaction. These results suggest greater combination-associated viability reduction in the SQOR-high lines but, with only four lines per group and no validated cutoff, do not establish SQOR as a treatment-predictive biomarker.

In the independent tumor-growth and terminal-weight cohort (n = 6 mice per group), mean day-28 tumor volumes were 1452 ± 231 mm^3^ in the vehicle group, 892 ± 176 mm^3^ with gemcitabine, 1008 ± 193 mm^3^ with the putative SQOR-directed compound, and 472 ± 126 mm^3^ with the combination. The combination reduced day-28 tumor volume relative to gemcitabine alone (raw P = 0.0013; Tukey-adjusted P = 0.0040). Corresponding terminal tumor weights were 1.52 ± 0.25, 0.91 ± 0.18, 1.03 ± 0.21, and 0.46 ± 0.13 g, respectively, with a lower terminal weight for the combination than for gemcitabine alone (raw P = 0.0019; Tukey-adjusted P = 0.0060). Values are mean ± SD. Despite growth suppression, tumor regression was not observed. In the independent survival cohort (n = 7 mice per group), median survival was 28 days for vehicle, 35 days for gemcitabine, 33 days for the putative SQOR-directed compound, and 49 days for the combination. Survival was prolonged with combination treatment relative to gemcitabine alone (raw log-rank P = 0.0071; Holm-adjusted P = 0.0210; Figure 7D). These efficacy observations do not establish that the compound acted through SQOR inhibition or that ferroptosis mediated tumor control.

## 4 Discussion

This study combines exploratory clinical associations with experimental evidence supporting a contribution of hypoxia-responsive SQOR to mitochondrial CoQ redox control, ferroptosis defense, and gemcitabine resistance in the tested PDAC models. In the 102-patient cohort, SQOR protein H-scores showed moderate positive associations with CAIX and HIF-1α (ρ = 0.473 and 0.438) and an inverse association with 4-HNE (ρ = −0.465). The SQOR-high group also had a lower exploratory mixed-endpoint response proportion than the SQOR-low group (29.8% vs. 54.5%) and a shorter median overall survival (14.2 vs. 23.8 months). In the prespecified clinicopathological Cox model, SQOR-high expression was associated with a mortality hazard ratio of 2.14; further adjustment for GPX4 and FSP1 attenuated, but did not eliminate, this association (hazard ratio, 1.87). The moderate tissue correlations support clinical associations but do not establish a hypoxia–SQOR–lipid-peroxidation causal pathway in patients. In particular, CAIX showed a weak inverse association with 4-HNE (ρ = −0.291; q = 0.00891955), whereas the HIF-1α–4-HNE association did not remain significant after FDR correction (ρ = −0.201; q = 0.0795635). Thus, the tissue data do not support a consistent inverse association between both hypoxia markers and 4-HNE. The heterogeneous associations between hypoxia markers and 4-HNE do not establish a hypoxia-mediated reduction in lipid peroxidation, which cannot be inferred from their separate correlations with SQOR. Lower 4-HNE staining is compatible with lower lipid-peroxidation burden, but does not by itself measure ferroptotic death. The hypoxia-exposure, SQOR-perturbation, CoQ-redox, lipid-peroxidation, and rescue experiments provide separate support for an SQOR‐dependent protective mechanism in the tested models. The tissue correlations do not validate this mechanism or establish causal mediation in human tumors.

The catalytic-mutant experiments were central to this interpretation. Although the catalytically inactive SQOR mutant was expressed at a level comparable to that of wild-type SQOR, it phenocopied SQOR knockdown, supporting a requirement for catalytic competence rather than protein abundance alone. Mutant effects on folding, localization, or protein interactions are not excluded by matched expression levels. At 24 h of hypoxia, the CoQH_2_/total CoQ ratio was approximately 1.6-fold above the normoxic baseline; knockdown or catalytic inactivation reversed this shift and increased both whole-cell and mitochondrial lipid ROS. This result is consistent with biochemical studies showing that human SQOR oxidizes H_2_S and transfers the resulting electrons to CoQ (Jackson et al., 2012; Landry et al., 2017), as well as with the broader view of SQOR as a regulator of mitochondrial sulfur and redox metabolism (Peng et al., 2025). The donor experiments further refined this mechanism. H_2_S donors increased the CoQH_2_ ratio and reduced lipid ROS, whereas inhibition or silencing of CBS and CSE blunted the hypoxia-induced CoQ shift. Selenium supplementation and perturbation had smaller effects that did not reach significance. Lee et al. (2024) demonstrated that hydrogen selenide can donate electrons through SQOR to ubiquinone and protect against ferroptosis before new selenoprotein production. The larger responses to H_2_S-pathway perturbation in Table 4 therefore describe the tested PDAC conditions, rather than contradicting that selenium mechanism or establishing a universal donor preference. Both catalytic inhibition and reduced SQOR abundance could decrease enzymatic output; these experiments do not establish the therapeutic superiority of either strategy.

The lipidomic results associate the CoQ redox shift with a ferroptosis-related phospholipid signature. SQOR knockdown and catalytic inactivation increased relative signals assigned to oxygenated AA- and AdA-containing PEs, whereas wild-type overexpression produced the opposite pattern. Oxidized PE-AA and PE-AdA species have been identified as proximal ferroptotic death signals (Kagan et al., 2017), and their abundance is strongly influenced by lipid remodeling pathways that load membranes with oxidation-prone polyunsaturated fatty acids or, conversely, protective monounsaturated fatty acids (Doll et al., 2017; Magtanong et al., 2019). The relative lipidomic pattern is consistent with this biochemical framework, but does not by itself exclude broader redox effects or confirm the structures and oxidation sites of individual lipids. It also places SQOR within a growing framework in which mitochondrial metabolism controls ferroptosis through the CoQ/CoQH_2_ pool. For example, perturbing mitochondrial complex I can induce mitochondrial ferroptosis through altered CoQH_2_ control (Deng et al., 2025), and recent syntheses emphasize that electron transport, CoQ metabolism, lipid remodeling, and compartment-specific antioxidant systems jointly determine ferroptosis sensitivity (Ding X. et al., 2025; Zheng and Conrad, 2025). Our data are consistent with sulfide oxidation contributing reducing power to the mitochondrial CoQ pool in the tested hypoxic PDAC models; the donor-perturbation assays do not directly quantify electron flux.

The epistasis experiments position SQOR as a partially independent component of the ferroptosis-defense network rather than an entirely new or fully separate pathway. Positive Bliss-excess values were confined mainly to intermediate-to-high perturbation regions, whereas responses at lower perturbation levels were additive or antagonistic. Chou–Talalay analysis likewise showed dose-dependent synergy: combination-index values were 0.68 and 0.61 for shSQOR plus RSL3 at ED50 and ED75, 0.74 and 0.65 for shSQOR plus iFSP1, 0.52 and 0.44 for shSQOR plus brequinar, and 0.57 at ED50 for shSQOR plus BAY 2402234. Wild-type SQOR overexpression rescued approximately 40% and 38% of the lipid-ROS increase in GPX4-and FSP1-deficient cells, respectively, but only about 18% in DHODH-deficient cells. The incomplete rescue is consistent with partially overlapping defenses. Convergence at mitochondrial CoQ is a plausible explanation for the DHODH results, but synergy and incomplete rescue do not uniquely define pathway topology; brequinar’s reported SQOR activity is an additional interpretive constraint (Jackson et al., 2022). This interpretation is consistent with the established compartmental organization of ferroptosis control: GPX4 directly reduces phospholipid hydroperoxides (Yang et al., 2014), FSP1 regenerates CoQH_2_ mainly at non-mitochondrial membranes (Bersuker et al., 2019; Doll et al., 2019), and DHODH supplies a mitochondrial CoQ-dependent defense (Mao et al., 2021).

Death-mode rescue supported a substantial ferroptotic contribution to the combination-associated loss of viability. Ferrostatin-1 and deferoxamine increased final viability from approximately 27%–31% to 62%–70% of untreated control, whereas Z-VAD-FMK produced only a small increase to approximately 30%–34%. These rescue effects cannot be translated into precise, mutually exclusive percentages of ferroptotic and apoptotic death. Badgley et al. (2020) similarly combined ferrostatin-1/DFO rescue with lipid-oxidation measurements in cysteine-depleted PDAC cells. The corresponding logic here is convergence of genetic perturbation, lipid-peroxidation readouts, and rescue, rather than numerical assignment of all lost viability to one death mechanism. Direct death measurements would further strengthen attribution. Even so, SQOR should be viewed as one node in a layered resistance network. In PDAC, stromal IL-15 can activate IL15RA–STAT3 signaling and increase GPX4 and ACSL3 (Wu et al., 2024), while tumor-cell-intrinsic SREBP1 and PITX2 programs can reinforce GPX4-dependent protection (Cao et al., 2024; Wang et al., 2024). SIK1 has also been linked to SLC7A11-dependent ferroptosis resistance through HDAC5–STAT6 signaling (Zhang H. et al., 2025), and epigenetic suppression of the NRF2–SLC40A1 axis can increase intracellular iron, induce ferroptosis, and enhance immunotherapy responses (Zhang Y. et al., 2025). These studies illustrate the diversity of iron, cystine, lipid, and stromal inputs that regulate ferroptosis in PDAC. The SQOR–CoQ axis adds a mitochondrial sulfur-oxidation component to this network but does not replace the established pathways.

The therapeutic experiments support further preclinical evaluation of combination treatment rather than SQOR-targeted treatment alone. Gemcitabine-resistant PANC-1 cells had approximately 2.8-fold more SQOR protein, a rise in the CoQH_2_/total CoQ ratio from about 27% to 49%, and baseline lipid ROS at roughly one-half of the parental level. The putative SQOR-inhibitor treatment reduced gemcitabine IC_50_ values in both resistant cell lines, but residual resistance remained 4.4-fold in PANC-1-GEM-R and 4.7-fold in MIA PaCa-2-GEM-R relative to their parental counterparts. Adding a ferroptosis inducer produced an overall six- to sevenfold reduction in gemcitabine IC_50_ relative to untreated resistant cells, yet the final values remained 2.0-fold and 1.9-fold above parental sensitivity. Thus, the intervention partially resensitized resistant cells but did not reverse resistance completely. This pattern is consistent with other PDAC studies in which altered lipid metabolism or stromal signaling couples ferroptosis avoidance to gemcitabine resistance. ACOT8, for example, changes cholesterol-ester and PUFA-containing phosphatidylethanolamine metabolism and promotes an antiferroptotic, gemcitabine-resistant state (Li B.-R. et al., 2025), whereas cancer-associated fibroblasts can stabilize SLC3A2 through an FGF1–PAX6–PAK3 pathway and thereby reinforce both ferroptosis resistance and gemcitabine resistance (Chen et al., 2026). Conversely, hypoxia-responsive engineered bacteria that deplete cysteine illustrate a distinct preclinical strategy for dismantling PDAC ferroptosis defenses (Qiao et al., 2025). These approaches reinforce the broader rationale for combining ferroptosis modulation with established PDAC therapies, while also underscoring that the field remains preclinical (Yang et al., 2024).

The organoid and xenograft results provide exploratory translational evidence. Among eight independently derived PDO lines, the four SQOR-high models showed paired viability reductions of approximately 46–52 percentage points when the SQOR inhibitor was added to gemcitabine, whereas the SQOR-low models showed smaller, heterogeneous changes. This descriptive treatment-by-biomarker pattern motivates testing of SQOR-based enrichment, but an eight-line, median-split analysis cannot establish a predictive cutoff. Tiriac et al. (2018) profiled a library of 66 PDAC organoid cultures and integrated functional and molecular information, illustrating a broader basis for pharmacotyping than the eight-line SQOR subgroup comparison here. PDOs can retain patient-specific heterogeneity (Beutel et al., 2025), and recent work has shown that pancreatic cancer organoid responses can align with clinical chemotherapy measures while also enabling autologous immune-cell testing (Anderko et al., 2026). A larger, prospectively defined PDO series will therefore be needed to determine whether SQOR modifies treatment benefit beyond its association with baseline drug sensitivity. *In vivo*, the combination reduced day-28 tumor volume and terminal tumor weight in the separate growth cohort (n = 6 per group) and prolonged survival in the independent survival cohort (n = 7 per group), but it did not produce tumor regression. The separation of these cohorts avoids reuse of animals across endpoints, although their limited sizes require cautious interpretation.

The clinical results are best interpreted as exploratory associations in gemcitabine-treated patients. Among 252 CA19-9-assessable nab-paclitaxel/gemcitabine recipients in MPACT, 206 (82%) had any CA19-9 decline at week 8 and 40 (16%) had an unconfirmed RECIST complete or partial response by week 8 (Chiorean et al., 2016). The radiological proportion was an early unconfirmed response measure, and the biochemical criterion was any decline, unlike the ≥50% threshold used here. This difference supports complementary interpretation and limits the meaning of a pooled response endpoint. Fisher and Pearson tests gave concordant results for the same fixed grouping; neither resolves assessment-method differences or accounts for the data-dependent cutoff. Following the principles of REMARK, cutoff-dependent survival estimates should be regarded as conditional on marker selection, and adjustment for GPX4/FSP1 does not replace adjustment for clinical confounders (Altman et al., 2012). The event counts and proportional-hazards diagnostics do not address the potential optimism introduced by same-cohort cutoff selection. Continuous-marker analyses and validation repeating the selection process remain necessary to evaluate model stability and a clinical decision threshold.

Published SQOR pharmacology provides a relevant but distinct comparator. Jackson et al. (2022) reported SQOR-targeted inhibitor 1 (STI1), with an enzymatic IC_50_ of approximately 29 nM and competition with CoQ. At 10.5 µM, more than 350-fold above that SQOR IC_50_, STI1 reduced complex-I-associated NADH oxidation by 21.6% and DHODH activity by 22.4%. These high-concentration findings should be interpreted within the reported selectivity profile, rather than as evidence of comparable inhibition of all three enzymes at nanomolar exposure. This concentration-dependent profile highlights the importance of relating cellular exposure to biochemical potency and off-target activity. Further compound-specific characterization is needed to determine whether the combination responses observed here depend on SQOR inhibition.

Analytical and *in vivo* evidence require separate interpretation. Kagan et al. (2017) linked defined oxygenated AA- and AdA-containing PEs to ferroptotic signaling through redox lipidomics and genetic perturbation. Criscuolo et al. (2022) demonstrated an MS/MS workflow for oxidized phosphatidylcholines, cholesteryl esters, and triacylglycerols in plasma, illustrating the analytical requirements for modification-specific lipid annotation rather than directly validating PDAC PE species. These studies provide a framework for interpreting the putative oxidized-PE features observed here. The genetic and rescue results support biological coherence; they do not rule out *ex vivo* lipid oxidation or establish positional isomers. *In vivo*, Badgley et al. (2020) demonstrated tumor-selective ferroptosis using Slc7a11 deletion and cyst(e)inase in pancreatic cancer models. Those interventions act through cysteine availability and cannot establish SQOR target engagement in the present xenografts. Our tumor-growth, weight, and survival observations show combination efficacy. Measurements of tumor SQOR activity, CoQ redox status, and ferroptosis markers would help establish the mechanism underlying this response.

The study is limited by its retrospective single-center clinical cohort and by mechanistic experiments in two KRAS-mutant PDAC cell lines. Tissue hypoxia was represented by CAIX and HIF-1α staining rather than direct oxygen measurements, and cross-sectional H-scores cannot establish temporal ordering. The modest marker associations and the use of 4-HNE as a surrogate for lipid-peroxidation burden limit claims about hypoxia-driven ferroptosis suppression in patients. Agreement between these models supports reproducibility within the tested backgrounds, but does not establish coverage of PDAC genetic or metabolic diversity. The small PDO subgroup comparison is hypothesis-generating, and the xenografts establish neither a predictive marker threshold nor the mechanism of drug action. Incomplete resensitization also indicates residual resistance mechanisms. Broader genetic backgrounds, independent clinical cohorts, continuous-marker validation, and pharmacologically characterized SQOR interventions are the next steps needed to test generalizability.

## 5 Conclusion

The experimental findings support hypoxia-responsive, catalytically active SQOR as a contributor to mitochondrial CoQ/CoQH_2_ cycling, ferroptosis defense, and gemcitabine resistance in the tested PDAC models. In patient tissues, SQOR abundance is associated with hypoxia-marker expression and lower 4-HNE staining; these modest correlations do not establish that hypoxia reduces lipid peroxidation through SQOR in patients. The clinical association remained significant across the two prespecified Cox models, and the cellular, epistasis, and rescue findings support SQOR as a contributor to ferroptosis defense in the tested models. Current efforts to improve PDAC care increasingly combine molecular stratification, functional testing, and rational multidrug strategies (Dusetti et al., 2025). The present findings provide a mechanistic rationale for evaluating the SQOR–CoQ axis within that framework through prospective clinical studies and further preclinical characterization of SQOR-directed interventions.

## Data Availability

The raw data supporting the conclusions of this article will be made available by the authors, without undue reservation.
